# Radiation Dose, Outcomes, and Trends of CT-Guided Osteoid Osteoma Ablation in Pediatric Patients: An Analysis of the Registry of the German Society for Interventional Radiology and Minimally Invasive Therapy

**DOI:** 10.1007/s00270-026-04412-8

**Published:** 2026-03-24

**Authors:** Charlotte Wintergerst, Michael Doppler, Sebastian Wirtz, Thomas Stein, Till Schürmann, Katharina Vogt, Elif Can, Jonathan Nadjiri, Wibke Uller

**Affiliations:** 1 Department of Diagnostic and Interventional Radiology, Division of Interventional Radiology, Medical Center – University of Freiburg, Faculty of Medicine, University of Freiburg, Hugstetterstr. 55, 79106 Freiburg, Germany; 2 Department of Interventional Radiology, School of Medicine and Klinikum Rechts Der Isar, Technical University of Munich, Munich, Germany

**Keywords:** Osteoid Osteoma, Pediatric, Microwave ablation, Radiofrequency ablation, Cryoablation

## Abstract

**Purpose:**

This study evaluated radiation dose parameters, clinical outcomes, and national trends in conventional CT-guided pediatric osteoid osteoma ablation.

**Materials and Methods:**

We retrospectively analyzed data from the German Society for Interventional Radiology and Minimally Invasive Therapy (DeGIR) registry (2019–2024). Patients ≤ 18 years with available Dose Length Product were included. Radiation dose, clinical outcome, and ablation modality were assessed. Benchmarks were defined using percentiles stratified by age and anatomical location.

**Results:**

A total of 166 pediatric patients (mean age 13.45 years; 59 female) were included. The median Dose Length Product was 132.00 (Q1: 53, Q3: 314) mGycm, 222.00 (Q1: 64.50, Q3: 485) mGycm for axial skeleton, and 124.00 (Q1: 51, Q3: 297) mGycm for extremity ablations. Technical success was 99.4% (partial technical success 0.60%), early clinical success was 58.43% (39.76% not assessable, 1.81% no improvement), and the complication rate was 1.81%. After additional age stratification and exclusion of very small groups (n < 5), Dose Length Product Q1; Q3 values were: axial skeleton 10–14 years 53; 504 mGycm (n = 6), 15–18 years 71; 483 mGycm (n = 17); extremities 5–9 years 55; 184 mGycm (n = 19), 10–14 years 43; 256 mGycm (n = 58), 15–18 years 50; 370 mGycm (n = 61). Radiofrequency ablation was most frequently applied (71.08%), followed by microwave ablation (26.51%), cryoablation (1.81%), and others (0.60%).

**Conclusion:**

This multicenter analysis provides proposed pediatric benchmarks for radiation exposure during conventional CT-guided osteoid osteoma ablation, which demonstrated excellent safety and technical success, supporting its role as standard therapy.

**Graphical Abstract:**

**Figure:**
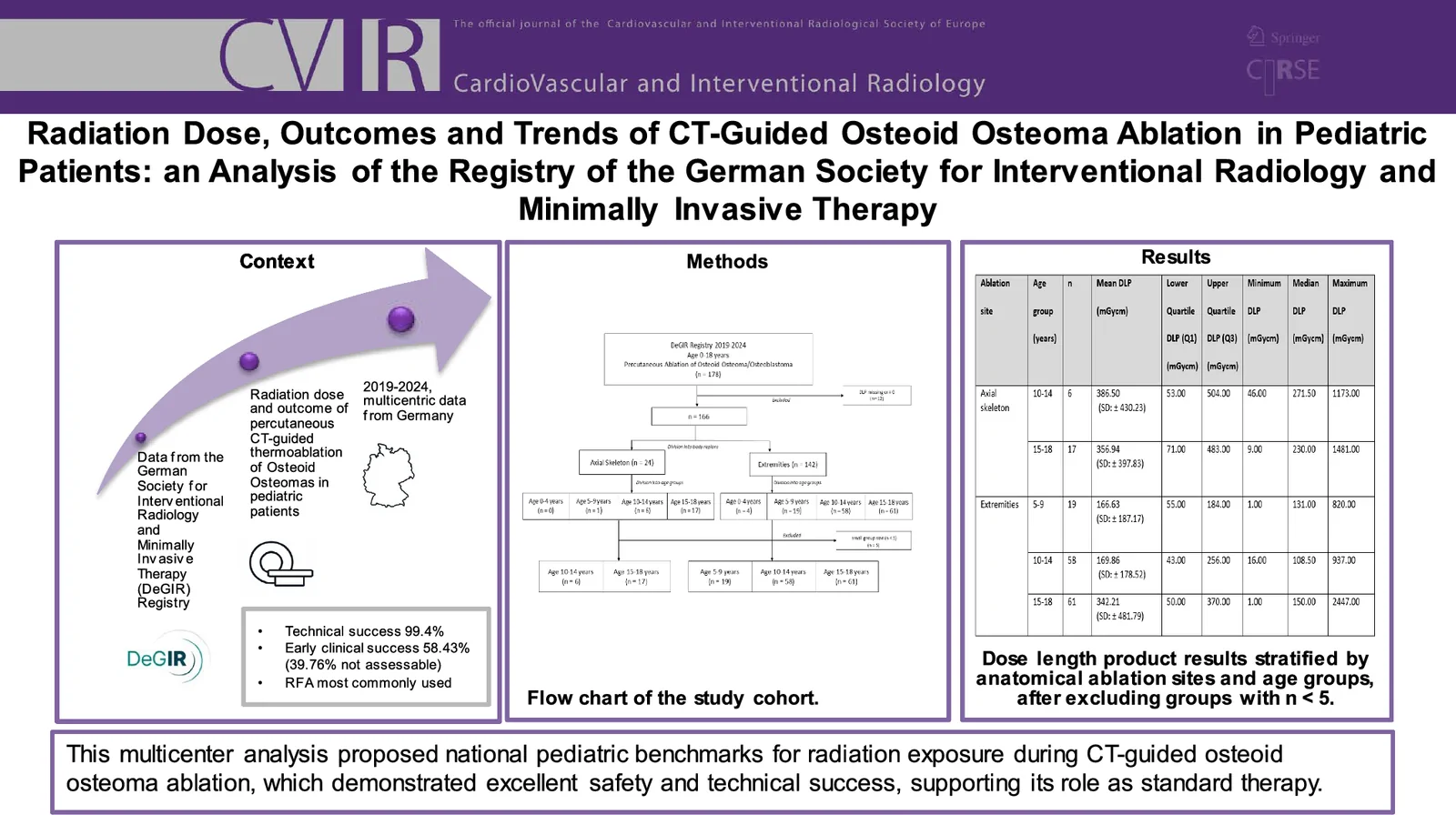

**Supplementary Information:**

The online version contains supplementary material available at https://doi.org/10.1007/s00270-026-04412-8.

## Introduction

Osteoid osteoma (OO) is a benign, painful tumor affecting most commonly long bones in patients with a peak presentation before the age of 20 years [1]. OOs account for 11% of benign bone tumors and 5% of all bone tumors [2]. Historically, patients with chronic pain refractory to conservative treatment have been treated surgically; however, there has been a shift from surgery toward percutaneous CT-guided thermal ablation (TA) as a standard therapy with high success rates and low complication rates [3–6]. While new approaches such as cone-beam CT have been described for OO ablation with low radiation exposure [7–9], conventional CT-guidance remains the imaging modality of choice in many centers due to its wide availability, and therefore, this study exclusively focused on procedures performed under conventional CT-guidance. RFA using alternating current to generate heat for the treatment of OO was introduced by Rosenthal in 1992 [10]. Since then, MWA, using electromagnetic waves to generate heat, and cryoablation (CA) using rapid freezing and thawing cycles have been introduced in the treatment of OO in 2007 and 2014, respectively [11–13].

Due to physiological and developmental factors, pediatric patients exhibit increased sensitivity to ionizing radiation compared to adults [14–16]. Diagnostic reference levels (DRLs) are key guidance for diagnostic and interventional radiological procedures [17, 18]. The application of DRL in clinical practice ensures procedural standardization and reduces unnecessary radiation exposure. This leads to improved long-term health outcomes, especially in pediatric patients. At the European level, DRLs for interventional radiological procedures in the pediatric population have not yet been defined. Similarly, at the national level in Germany, DRLs for CT-guided interventions are currently unavailable for both adult and pediatric patients [19, 20]. Furthermore, studies that combine the analysis of radiation dose of CT-guided TA of OOs with its clinical and technical outcome and complication data remain scarce.

The purpose of this multicenter study was 1) to evaluate radiation dose parameters during CT-guided ablation of OOs in pediatric patients using data from the German Society for Interventional Radiology and Minimally Invasive Therapy (DeGIR) registry collected between 2019 and 2024, 2) to propose benchmarks as stratified by age groups and body regions, and 3) to assess technical and early clinical success rates, complication rates, and trends in the use of different ablation techniques over time.

## Materials and Methods

### Study Design and Subjects

This retrospective, multicenter registry study was carried out in accordance with the Declaration of Helsinki. It was registered in the German Clinical Trials Register (DRKS) under the identifier DRKS00033244. Approval was granted by the local ethics committee at the University of Freiburg, Germany (reference: 24–1001-S1-retro).

Data sets of patients under the age of 19 years on percutaneous OO/osteoblastoma ablation from 2019 until 2024 were obtained from the registry of the German Society for Interventional Radiology and Minimally Invasive Therapy (DeGIR) (Samedi GmbH, Berlin, Germany) and included. Participating centers may voluntarily submit data on image-guided procedures through a standardized web-based reporting form (**See supplementary material 1)**. Per-procedure radiation exposure was quantified using the total Dose Length Product (DLP) summed over all CT acquisitions according to Yang et al. [21]. CTDI (Computed Tomography Dose Index) was not analyzed, as its definition is not well suited for CT-guided interventions with stationary table acquisitions and repeated imaging of the same region. In addition, information on the specific acquisition modes corresponding to the reported CTDI values was not available from the registry.

Exclusion criteria were a DLP = 0 mGycm or missing data on DLP.

The analysis included the patient’s age, gender, ablation modality (RFA, MWA, CA), and DLP. The variables “technical success” and “early clinical success” were evaluated using a structured questionnaire with predefined response options. The DeGIR provided definition examples (**Supplementary material 1).** An example of the definition of technical success was a full ablation protocol being carried out upon correct probe positioning. Participating centers were also given the option to report partial technical success (defined as a tumor completely ablated, but without an adequate safety margin). Early clinical success was defined as pain reduction immediately following the intervention, with response categories “yes,” “no,” and “not assessable”; no specific post-interventional time frame was indicated in the DeGIR questionnaire. The variable “protective organ displacement” was included to define thermoprotective procedures. The distribution of OOs ablation procedures was stratified by federal state.

Complications were self-reported according to the guidelines of the Society of Interventional Radiology (SIR) (**Supplementary material 1**) [22]. For reporting purposes, improved comparability, and conceptual overlap, we applied a predefined rule-based mapping from SIR to Cardiovascular and Interventional Radiological Society of Europe (CIRSE) grades reflecting the clinical impact and required therapy. Grade 1 describes events with no need for therapy and no consequences, and Grade 2 covers complications that require nominal therapy but do not prolong hospital stay. Grade 3 includes events that require major therapy or lead to prolonged hospitalization without permanent sequelae. Higher grades (4–6) indicate life-threatening complications, permanent sequelae, or death [23].

### Statistical Analysis

Statistical analysis was performed using the Statistical Analysis System (SAS, Version 9.4). The data obtained from the DeGIR registry are presented as arithmetic means with standard deviations (SD) for continuous variables or as counts and percentages for categorical variables. The mean, median, minimum (min), maximum (max), SD, and the 25th and 75th percentiles (Q1 and Q3), respectively, were calculated and proposed as benchmarks.

In the first step, patients were grouped according to their anatomical tumor location. Anatomical regions were categorized as follows: (1) Spine, thorax, and pelvis were classified collectively as the “axial skeleton,” and (2) upper and lower limbs (including femur) were classified collectively as the “extremities.” In a second step, the patients were stratified according to age groups: 0–4 years, 5–9 years, 10–14 years, and 15–18 years, in accordance with the approach proposed by Schegerer et al. [19]. This stratification was selected because the registry provided only the year of birth and the date of the procedure, but not the exact age at the time of intervention, and because broader age categories were necessary to ensure sufficiently sized strata for this rare interventional procedure. Groups consisting of fewer than 5 patients (n < 5) were excluded to define final benchmarks. We report the mean and 25th and 75th percentiles (Q1 and Q3) of the DLP distribution as a procedure-specific benchmark range for each age group and anatomical region. These benchmark ranges are intended as orientation values for local quality assurance and optimization rather than as formal DRLs.

## Results

### Study Population

**Fig. 1:**
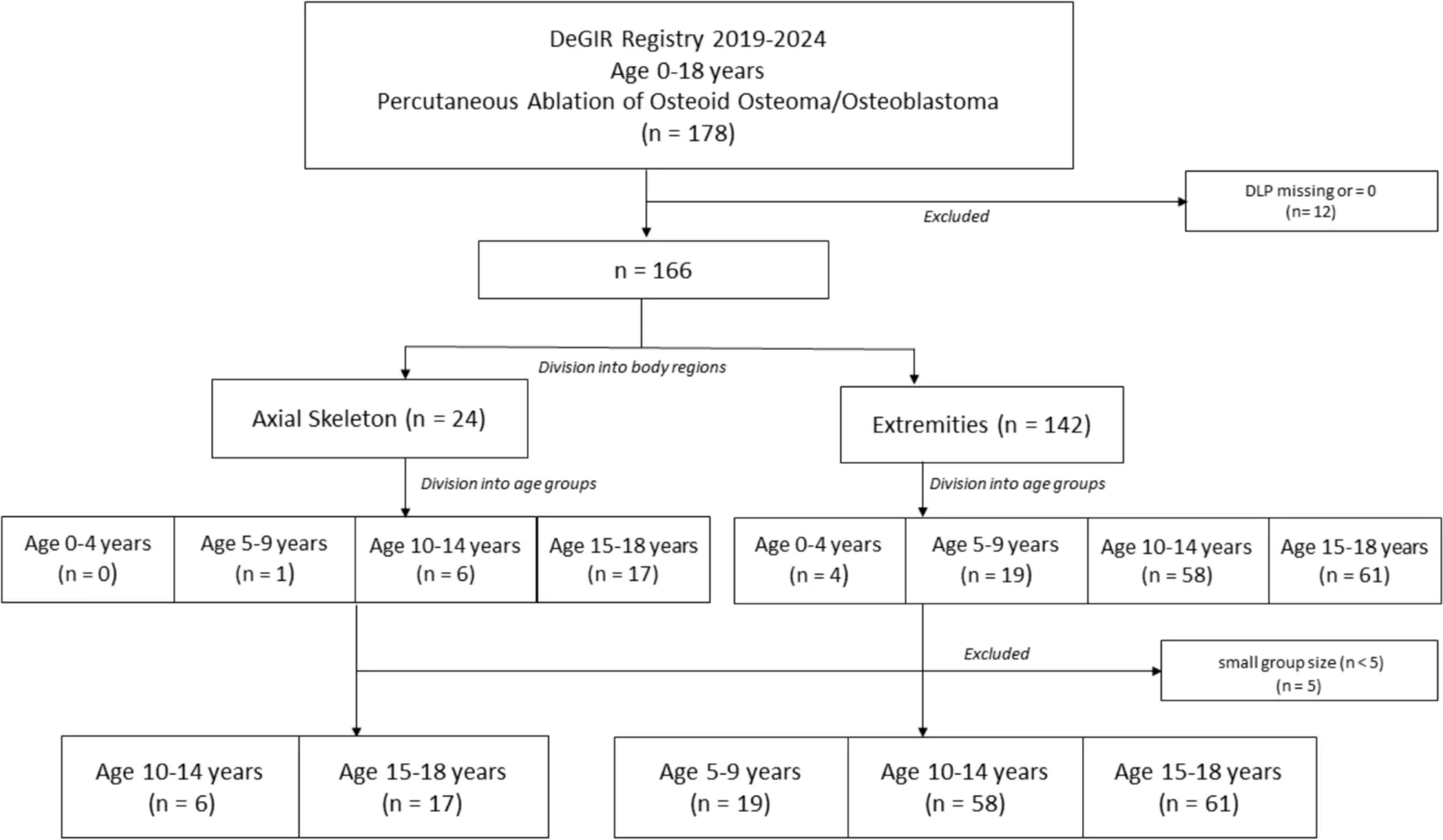
Flowchart of the study cohort. After application of inclusion criteria, patients were stratified according to anatomical region (“axial skeleton” vs. “extremities”) and age group (0–4, 5–9, 10–14, 15–18 years). Groups with n < 5 patients were excluded. *DeGIR* German Society for Interventional Radiology and Minimally Invasive Therapy, *DLP* Dose length product

A total of 178 patients under 18 years of age who underwent CT-guided ablation of an OO/osteoblastoma were identified in the DeGIR Registry from 2019 to 2024 **(**Fig. 1**).** A total of 12 patients were excluded (missing or DLP = 0), yielding a final cohort of 166 children and young adults (59 female) included in the analysis.

The mean age of the included patients was 13.45 years (SD: ± 3.84 years, median: 14 years, range: 0–18 years)**.** Characteristics of the study population are shown in Table 1**.** OO ablations were performed across multiple federal states, with the highest numbers observed in Baden-Württemberg (n = 39, 23.5%), North Rhine–Westphalia (n = 32, 19.3%), and Bavaria (n = 26, 15.7%) (Supplementary material 2, Supplementary Table 1).

**Table 1:** Characteristics and outcomes of the study cohort.

	n = 166	Ablation of axial skeleton n = 24	Ablation of extremities n = 142
Age (years)	13.45 (SD: ± 3.84)	15.04 (SD: ± 1.94)	13.18 (SD: ± 4.02)
Gender	59 (35.54%) female	4 (16.67%) female	55 (38.73%) female
	107 (64.46%) male	20 (83.33%) male	87 (61.27%) male
Cryoablation	3 (1.81%)	1 (4.17%)	2 (1.41%)
Radiofrequency ablation	118 (71.08%)	18 (75%)	100 (70.42%)
Microwave ablation	44 (26.51%)	5 (20.83%)	39 (27.46%)
Other	1 (0.60%)	0	1 (0.70%)
Dose length product (mGycm)	262.59 (SD: ± 359.15)	358.21 (SD: ± 389.24)	246.43 (SD: ± 352.72)
Technical success	Yes 165 (99.40%)	Yes 24 (100%)	Yes 141 (99.30%)
	Partially 1 (0.60%)	Partially 0	Partially 1 (0.70%)
Early clinical success	Yes 97 (58.43%)	Yes 11 (45.83%)	Yes 86 (60.56%)
	No 3 (1.81%)	No 3 (12.5%)	No 0
	Not assessable 66 (39.76%)	Not assessable 10 (41.67%)	Not assessable 56 (39.44%)
Complications	No 163 (98.19%)	No 23 (95.83%)	No 140 (98.59%)
	Grade 1, 1(0.60%)	Grade 3, 1 (4.17%)	Grade 1, 1(0.70%)
	Grade 2, 1(0.60%)		Grade 2, 1(0.70%,)
	Grade 3, 1(0.60%)		
Protective Organ displacement	Yes 1 (0.60%)	Yes 1 (4.17%)	Yes 0 (0.00%)

Data are means with standard deviation or numbers with percentages. SD—Standard deviation

The axial skeleton was targeted for ablation in 24 patients (4 female), and the extremities were ablated in 142 patients (55 female). In the second step, stratification by age resulted in the following distribution: 1. for the axial skeleton age 0–4 years (n = 0), age 5–9 years (n = 1, 0 female), age 10–14 years (n = 6, 2 female), age 15–18 years (n = 17, 2 female), and 2. for the extremities age 0–4 years (n = 4, 2 female), age 5–9 years (n = 19, 8 female), age 10–14 years (n = 58, 16 female), and age 15–18 years (n = 61, 29 female).

### Dose Length Product and Proposed Benchmarks

The overall median DLP was 132.00 mGycm (SD: ± 359.15, mean 262.59, min 1.00 mGycm, max 2447.00 mGycm). The lower quartile (Q1) measured 53.00 mGycm, while the upper quartile (Q3) measured 314.00 mGycm. Stratified only to ablations of the axial skeleton, the median DLP measured 222.00 mGycm (SD: ± 389.24 mGycm, mean 358.21 mGycm, min 9.00 mGycm, max 1481.00 mGycm), with a Q1 of 64.50 mGycm and a Q3 of 485.00 mGycm, while the ablations of the extremities measured a median of 124.00 mGycm (SD: ± 352.72 mGycm, mean 246.43 mGycm, min 1.00 mGycm, max 2447.00 mGycm) and a Q1 of 51.00 mGycm and a Q3 of 297.00 mGycm.

**Fig. 2:**
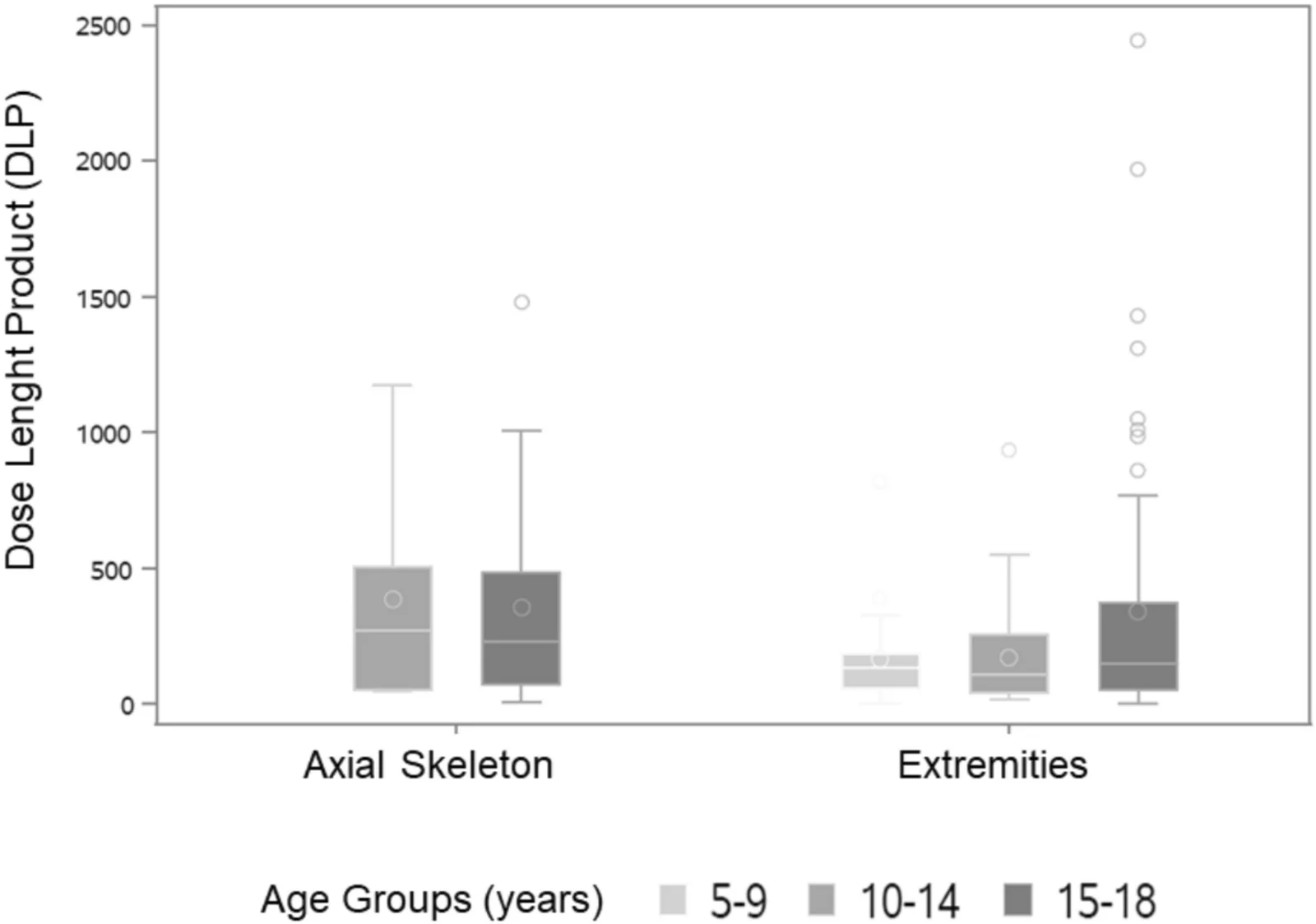
Boxplots of Dose Length Product stratified by anatomical region (axial skeleton vs. extremities) and age group (5–9, 10–14, and 15–18 years). The boxes represent the interquartile range with the horizontal line indicating the median; whiskers extend to the smallest and largest values within 1.5 times the interquartile range from the lower and upper quartiles, respectively. Circles represent outliers

The results and proposed benchmarks (Q1, Q3) obtained after further stratification into defined age groups and exclusion of 5 patients due to a small group (n < 5) are shown in Table 2**,** Boxplots of DLP stratified by anatomical region are shown in Fig. 2. Data were assessed for skewness and were found to be right-skewed.

**Table 2:** Dose Length Product results stratified by anatomical ablation sites and age groups, after excluding groups with n < n. Q1 and Q3 represent proposed benchmarks

Ablation site	Age group (years)	n	Mean DLP (mGycm)	Lower Quartile DLP (Q1) (mGycm)	Upper Quartile DLP (Q3) (mGycm)	Minimum DLP (mGycm)	Median DLP (mGycm)	Maximum DLP (mGycm)
Axial skeleton	10–14	6	386.50 (SD: ± 430.23)	53.00	504.00	46.00	271.50	1173.00
	15–18	17	356.94 (SD: ± 397.83)	71.00	483.00	9.00	230.00	1481.00
Extremities	5–9	19	166.63 (SD: ± 187.17)	55.00	184.00	1.00	131.00	820.00
	10–14	58	169.86 (SD: ± 178.52)	43.00	256.00	16.00	108.50	937.00
	15–18	61	342.21 (SD: ± 481.79)	50.00	370.00	1.00	150.00	2447.00

DLP—Dose Length Product, SD—Standard deviation

### Outcomes

The overall technical success rate was 99.40% (n = 165); a partial technical success was achieved in 0.60% (n = 1) of patients. The early clinical success rate was 58.43% (n = 97), while 1.81% (n = 3) showed no improvement; in 39.76% (n = 66) of patients, the outcome was reported as not assessable.

Protective organ displacement was used in one patient, while in 165 patients, there was no data entry. Complications occurred in 3 of 166 procedures (1.81%): one (0.60%, Grade 3 according to CIRSE) within the first 24 h after ablation and two (1.20%, Grade 1 and Grade 2) beyond the first 24 h post-intervention. Table 1 shows the outcomes stratified by ablation site.

### Ablation Techniques

**Fig. 3:**
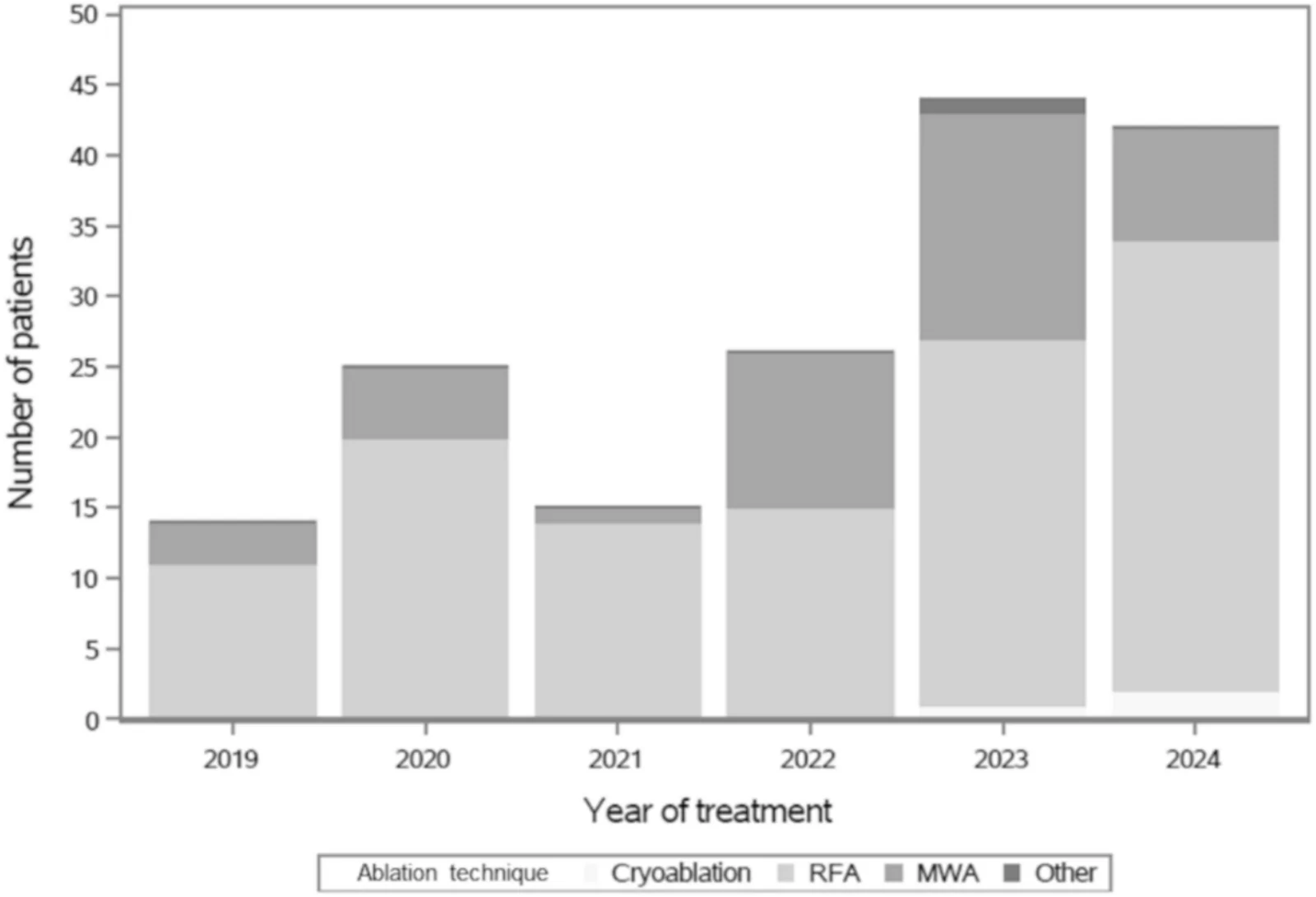
Use of different thermal ablation techniques for the treatment of pediatric osteoid osteomas. Data from the DeGIR registry, 2019–2024. DeGIR—German Society for Interventional Radiology and Minimally Invasive Therapy

We observed an overall increase in the number of CT-guided TAs for OOs performed over the years, except in 2021, and a plateau in 2023–2024 **(**Fig. 3**).**

RFA was the most commonly used ablation technique (71.08%, n = 118), followed by MWA (26.51%, n = 44), CA (1.81%, n = 3), and others (0.60%, n = 1).

RFA demonstrated a consistently high share over the years, peaking at 93.33% in 2021 and remaining the predominant technique thereafter. MWA usage fluctuated without a clear upward trend, whereas cryoablation, introduced in 2023, was used for 3 patients (2023: 1, 2024: 2).

## Discussion

We analyzed the radiation dose during CT-guided ablation of OOs in pediatric patients using multicentric, national DeGIR registry data (2019–2024), with a focus on anatomical and age-specific differences. We established proposed benchmarks and evaluated procedural outcomes and trends in ablation techniques. In the absence of established national or international DRL or guideline recommendations for CT-guided OO ablations in pediatric patients, these values provide study-based orientation for quality assurance and protocol optimization, rather than as formal DRLs. Our findings provide clinicians with valuable evidence to support informed discussions with parents about OO ablation, particularly when weighing the risks of radiation exposure against the expected outcome. Furthermore, we demonstrated the value of the DeGIR registry as a platform for generating the necessary evidence in the future.

Previous small-scale retrospective studies analyzed the DLP of CT-guided OO ablations in children. Fiore et al. reported in 2022 a mean DLP of 972.62 (range 337–2344) mGycm in 29 children and adolescents, while Perry et al. reported in 2017 a mean DLP of 61.5 (range 23.4–143.5) mGycm in 35 pediatric patients [7, 9]. Cheng et al. found in 2014 average DLPs of 1058.83 mGycm for CT-guided OO ablation in 36 pediatric and adult patients (mean age 19 years (range 7–52)) [8]. While the focus of our study was on quartiles, the mean DLP measured 262.59 (range 1–2447) mGycm in 166 children, lower than reported by Fiore et al. and Cheng et al., but higher than by Perry et al. Differences in mean DLP values should be interpreted with careful consideration of the ablation site; in Perry et al.’s study, 33 out of 35 ablations were performed in the lower extremities and 2 in the pelvis [9]. The lower radiation dose in CT-guided TA of extremities stems from reduced scan length and the absence of dose-intensive structures. To enable more accurate analysis, we grouped the cohort by anatomical ablation sites into “axial skeleton” and “extremities.” Our mean DLP for 142 extremity ablations was 246.43 (range 1.00–2447) mGycm, higher than found by Perry et al. This may be attributable to the multicenter design of our study, larger patient population, and heterogeneous CT systems and protocols, which provide a more representative estimation of the actual radiation dose required.

Overall DLP for Q1 and Q3 was 53.00 and 314.00 mGycm, with values ranging from 1.00 to 2447.00 mGycm. The wide inter-center variability in DLP reflects differences in lesion location, accessibility, and in CT system characteristics and protocol settings. As per-procedure DLP in our study was defined as the sum across all CT acquisitions, these heterogeneous acquisition strategies inevitably result in a broad dose spectrum across centers. CTDI was not analyzed due to the limited applicability in CT interventions with a stationary table and repeated acquisitions, and missing information on acquisition modes in the registry.

Radiation exposure bears higher risks in the pediatric population [16, 24]. When counseling families, it helps to convert dose values to an effective dose and/or compare them with those from other CT examinations. We have deliberately refrained from doing so, as such comparisons depend heavily on the region of the body being examined and do not adequately reflect the actual dose distribution; moreover, effective dose is a population-based metric unsuitable for individual risk assessment [25]. We proposed benchmarks for quality control for physicians rather than legally binding thresholds or a direct basis for DRL, as essential parameters such as patient body size and standardized CT protocols were not collected.

Systematic reviews reported clinical success rates of 90–100% for CT-guided TA of OOs [4, 6]. In our study, early clinical success was achieved in 58.43%, not achieved in 1.81%, and not assessable in 39.76%, probably due to a lack of post-procedural follow-up at the time of data entry into the DeGIR registry, which may lead to an underreporting of the true success rate. Overall, we observed a technical success rate of 99.40% and a partial success rate of 0.60%. Previously, technical failure rates of 0.3% were reported [6]. In our study, the complication rate was 1.81%, which is in line with data from published literature (2–2.5%), pointing to an overall safety of CT-guided TA of OOs [4, 6].

With high success, minimal complications, and rare non-response, TA has become the standard therapy for OOs [4, 6]. CT-guided TAs increased over time, except in 2021, peaking in 2023. Unlike overall CT-guided TA procedures, which remained stable during the coronavirus 2019 (COVID-19) pandemic in Germany [26], OO CT-guided TAs declined sharply in 2021, reflecting their highly elective nature. RFA was the most frequently performed OO TA in our cohort, reflecting its early establishment and proven efficacy [5]. The second most performed TA was MWA, peaking in 2023. Despite being newer than RFA [12, 13], a systematic review from 2025 reports excellent clinical success rates and low complication rates [27]. CA for OOs reports clinical success rates ranging from 90.5% to 100%, with complication rates between 0 and 21% (all minor) [28, 29]. After its introduction in Germany in 2023, CA was only performed in 3 ablations (1.81% of all procedures over the years), and its future role in clinical practice remains to be determined.

Due lacking data about the participating centers, we report federal-state-level data, with Baden-Württemberg contributing the highest number of ablations, followed by North Rhine–Westphalia and Bavaria, likely reflecting concentration at specialized centers.

Our study has certain limitations; the data we gathered stem from the DeGIR registry based on voluntary participation, which may lead to a lack of or incomplete data. Optional reporting without central monitoring may introduce reporting bias, and lacking data about body size and weight hindered a body-size-adapted definition. Details on CT protocols, long-term clinical outcome data, and imaging are missing, and technical success could only be analyzed by a surrogate parameter. Due to the makeup of the DeGIR registry, we included ablation classified as OOs, as well as osteoblastomas from the DeGIR registry. The variables “early clinical success” and “technical success” were assessed using predefined response options. Although the DeGIR provided definition examples for “technical success,” it did not specify strict operational definitions. No strict post-procedural time frame was indicated for “early clinical success,” potentially allowing some degree of subjective interpretation and introducing reporting bias. Complications were self-reported and categorized according to SIR grades, so that a more granular CIRSE subclassification was not possible. Despite these limitations, the analysis of the DeGIR registry still provided important multicenter real-life data on CT-guided TA of OOs in a large pediatric cohort.

## Conclusion

We evaluated the DLP and proposed anatomic- and age-specific benchmarks of conventional CT-guided TA of OOs in children using DeGIR registry data from 2019 to 2024 without navigation. These benchmarks can help to minimize pediatric radiation exposure, hence improving patient safety and long-term patient outcomes; however, these benchmarks require further validation and cannot replace national and international DRLs. CT-guided TAs for OO are safe interventions with excellent outcomes. The overall increase in this technique reflects a shift from surgical to minimally invasive treatment.

## Supplementary Information

Below is the link to the electronic supplementary material.

Supplementary file1 (DOCX 20 KB)

Supplementary file2 (DOCX 17 KB)

## Data Availability

Restrictions apply to the availability of the data generated or analyzed during this study to preserve patient confidentiality or because they were used under license. The corresponding author will, on request, detail the restrictions and any conditions under which access to some data may be provided.

## Abbreviations

CA: Cryoablation
CIRSE: Cardiovascular and Interventional Radiological Society of Europe
CTDI: Computed Tomography Dose Index
DeGIR: German Society for Interventional Radiology and Minimally Invasive Therapy
DLP: Dose Length Product
DRL: Diagnostic reference levels
OO: Osteoid osteoma
TA: Thermal ablation
SD: Standard deviation
SIR: Society of Interventional Radiology
