# Supplementary material for: Radiation Dose, Outcomes, and Trends of CT-Guided Osteoid Osteoma Ablation in Pediatric Patients: An Analysis of the Registry of the German Society for Interventional Radiology and Minimally Invasive Therapy
Source: Cardiovasc Intervent Radiol. 2026 Mar 24;49(11):2408–17. doi: 10.1007/s00270-026-04412-8 (PMC13751765; doi:10.1007/s00270-026-04412-8)
Supplement: Supplementary file 1 [file 270_2026_4412_MOESM1_ESM.docx]

Supplementary material 1:

**Online form for data submission for image-guided interventions for the DeGIR registry**

(Samedi GmbH, Berlin, Germany)

Mandatory questions are marked with *.

Answering options are given in brackets.

**Patient data**

Sex* (male; female; diverse)

Date of birth*

**1 – Intervention**

Date*

Region* (skeletal system)

Target detail* (Extremity (Foot; Lower leg; Thigh; Hand; Forearm; Upper arm), Spine (Cervical spine; Thoracic spine; Lumbar spine; Sacrum / Coccyx), Pelvis (Ilium; Ischium; Pubis; Symphysis; Sacroiliac joint), Miscellaneous (Ribs; Shoulder girdle; Sternum; Joint))

Interventionalist

Interventionalist 1

Interventionalist 2

Interventionalist 3

Outpatient intervention?* (yes; no)

Begin of intervention outside routine daily shift* (yes; no)

**2 – Preinterventional diagnostic**

Therapeutic intention: (curative; palliative (symptom/mass reduction))

Laboratory values *

Quick/INR* (n.a.; normal; pathological)

PTT* (n.a.; normal; pathological)

Thrombocyte count* (n.a.; normal; pathological)

Creatinine/GFR* (n.a.; normal; pathological)

Preinterventional imaging *

X-ray (excl. mammography) (yes)

Mammography (yes)

Tomosynthesis (yes)

CT/CTA (yes)

MRI/MRA (yes)

DSA/Fluroscopy (yes)

Ultrasound (yes)

Scintigraphy/SPECT/SPECT-CT (yes)

PET/PET-CT/PET-MRI (yes)

not performed (yes)

Clinical indication *

Preoperative procedure? (yes; no)

Metastasis? (yes)

Primary malignant tumour? (yes)

Osteoid osteoma / Osteoblastoma? (yes)

Other (yes)

Unknown (yes)

Lesion size (mm)

Clinical symptoms (yes; no)

if yes:

Pain

Fracture

Neurological Deficit

Paraneoplastic syndrome

B-symptoms

Interdisciplinary conference *

Anaesthesiology (yes)

General surgery / Vascular surgery (yes)

Internal medicine (yes)

Neurosurgery (yes)

Neurology (yes)

Gynecology (yes)

Radiology (yes)

Pathology (yes)

Radiation therapy (yes)

Nuclear medicine (yes)

Urology (yes)

Others (yes)

Tumor Conference decision (yes)

Not performed (yes)

**3 – Description of intervention**

Anaesthesia *

None (yes)

Local (yes)

Sedation (yes)

Analgosedation (yes)

Intubation (yes)

High-flow ventilation (yes)

Anaesthesiological stand-by (yes)

Other (yes)

Medication

Antibiotic treatment (yes; no)

Method of ablation *

RFA (yes)

Laser (LTT) (yes)

Microwave ablation (yes)

Cryoablation (yes)

Focused ultrasound (yes)

Irreversible electroporation (IRE) (yes)

Electrochemotherapy (ECT) (yes)

Intraoperative (yes)

Other (yes)

Additional method

Additional ablation of the needle track (yes)

Protective organ displacement (e.g. using gas or liquid) (yes)

Cooling (yes)

Temporary vascular occlusion (e.g. balloon occlusion) (yes)

**4 – Quality of results**

Image-guided technique *

CT (yes)

CTDI vol*

DLP*

DSA/Fluroscopy (yes)

Cone beam CT (yes; no)

Duration (min)

DAP

MRI (yes)

Ultrasound (yes)

Mammography (yes)

Difficult conditions *

None (yes)

Obesity (yes)

Patient non-compliance (yes)

Ventilated patient (yes)

Increased risk for use of contrast agent (yes)

Cardiovascular instability (yes)

Immobilized patient (yes)

Difficult access (yes)

Difficult local tumor situation (yes)

Need of special technique (yes)

Other (yes)

Technical success

Technical success* (yes; no; partial success)

- Note – Examples of definitions of success :

Osteoid osteoma: Successful completion of the ablation protocol (with correct probe positioning)

Malignant tumor: Success: Tumor completely ablated with an adequate safety margin

Partial success: Tumor completely ablated without an adequate safety margin

Palliative indication: Successful completion of the ablation protocol (with correct probe positioning)

Curative indication: Tumor completely ablated with an adequate safety margin

Uterine fibroid: Successful completion of the ablation protocol

Clinical success early post-interventional

Clinical success early post-interventional* (yes; no; not assessable)

-Note: Osteoid osteoma: Pain reduction

Malignant tumor: Not assessable

Pain indication: Pain reduction

Curative indication: Not assessable

Uterine fibroid: Not assessable

Postinterventional imaging *

No imaging (yes)

X-ray (excl. mammography) (yes)

Mammography (yes)

CT/CTA (yes)

MRI/MRA (yes)

DSA (yes)

Ultrasound/Duplex (yes)

Scintigraphy/SPECT/SPECT-CT (yes)

PET/PET-CT/PET-MRI (yes)

**-**Note: Outcome assessment immediately after completion of the intervention.

**Complications Module A–D**

Complications Module A–D within 24h post intervention* (yes; no)

If yes:

arterial bleed

venous bleeding

arterial occlusion

venous occlusion

aneurysm

cardiac complication

neuro-cerebral complication

pulmonary complication

infection/abscess

dislocation of external material (primary or secondary)

parenchymal bleeding

tissue ischemia or infarction

anaphylaxia

organ dysfunction/failure

adverse medication reaction

organ injury

other

Grading of the complication:

A – No treatment required, no consequences

B – Symptomatic treatment, observation if necessary (overnight)

C – Treatment required, short hospitalization (< 48 h)

D – Higher level of treatment required, unplanned escalation of care, prolonged hospitalization (> 48 h)

E – Permanent damage

F – Death

Complication treated? (no; self; other discipline)

If yes: Success? (yes; no)

Complications Module A–D after 24h post intervention* (yes; no)

Termination of intervention (yes; no)

If yes: Reason

– technical/anatomical reason

– non-compliance of the patient

– complication independent of the intervention

– complication associated with the intervention

– technical failure of the machine

– probing of the lesion was not successful

– other

Multiple interventions? (yes)
