# Supplementary material for: Radiation Dose, Outcomes, and Trends of CT-Guided Osteoid Osteoma Ablation in Pediatric Patients: An Analysis of the Registry of the German Society for Interventional Radiology and Minimally Invasive Therapy
Source: Cardiovasc Intervent Radiol. 2026 Mar 24;49(11):2408–17. doi: 10.1007/s00270-026-04412-8 (PMC13751765; doi:10.1007/s00270-026-04412-8)
Supplement: Supplementary file 2 [file 270_2026_4412_MOESM2_ESM.docx]

| **Federal State** | **Frequency** | **Percentage (%)** |
| --- | --- | --- |
| Baden-Württemberg | 39 | 23.49 |
| North Rhine–Westphalia | 32 | 19.28 |
| Bavaria | 26 | 15.66 |
| Berlin | 14 | 8.43 |
| Lower Saxony | 13 | 7.83 |
| Saxony | 12 | 7.23 |
| Rhineland-Palatinate | 11 | 6.63 |
| Hesse | 10 | 6.02 |
| Saarland | 4 | 2.41 |
| Hamburg | 1 | 0.60 |
| Mecklenburg–Western Pomerania | 1 | 0.60 |
| Schleswig-Holstein | 1 | 0.60 |
| Thuringia | 1 | 0.60 |
| Austria | 1 | 0.60 |

Supplementary Table 1. Distribution of osteoid osteoma ablations included in the study by federal state.
